# Molecular Decolonization: An Indigenous Microcosm Perspective of Planetary Health

**DOI:** 10.3390/ijerph17124586

**Published:** 2020-06-25

**Authors:** Nicole Redvers, Michael Yellow Bird, Diana Quinn, Tyson Yunkaporta, Kerry Arabena

**Affiliations:** 1 Department of Family & Community Medicine, University of North Dakota School of Medicine & Health Sciences, Grand Forks, ND 58202, USA; 2 Arctic Indigenous Wellness Foundation, Yellowknife, NT, X1A 2N5, Canada; 3 InVIVO Planetary Health, of the Worldwide Universities Network (WUN), New York, NJ 10704, USA; 4 Faculty of Social Work, University of Manitoba, Winnipeg, MB R3T 2N2, Canada; Michael.YellowBird@umanitoba.ca; 5 Centre for Mindful Decolonization, University of Manitoba Faculty of Social Work, Winnipeg, MB R3T 2N2, Canada; drquinn@drdianaquinn.com; 6 National Indigenous Knowledge Research and Innovation (NIKERI) Institute, Deakin University, Waurn Ponds Campus, Waurn Ponds, VIC 3216, Australia; tyson.yunkaporta@deakin.edu.au; 7 First 1000 Days Australia, Riddells Creek, VIC 3431, Australia; kerryarabena@karabenaconsulting.com

**Keywords:** planetary health, Indigenous health, climate change, environmental health, ecology, health equity, knowledge translation, environmental stewardship, microbiome

## Abstract

Indigenous peoples are resilient peoples with deep traditional knowledge and scientific thought spanning millennia. Global discourse on climate change however has identified Indigenous populations as being a highly vulnerable group due to the habitation in regions undergoing rapid change, and the disproportionate burden of morbidity and mortality already faced by this population. Therefore, the need for Indigenous self-determination and the formal recognition of Indigenous knowledges, including micro-level molecular and microbial knowledges, as a critical foundation for planetary health is in urgent need. Through the process of Indigenous decolonization, even at the smallest molecular scale, we define a method back to our original selves and therefore to our planetary origin story. Our health and well-being is directly reflected at the planetary scale, and we suggest, can be rooted through the concept of molecular decolonization, which through the English language emerged from the ‘First 1000 Days Australia’ and otherwise collectively synthesized globally. It is through our evolving understanding of decolonization at a molecular level, which many of our Indigenous cultural and healing practices subtly embody, that we are better able to translate the intricacies within the current Indigenous scientific worldview through Western forms of discourse.

## 1. Introduction

*You cannot understand true medicine power (in whatever capacity that may be) unless you have an understanding of the nature of things. To understand the nature of things, you need to be in nature, and you need to connect to the animals, the rocks, the plants, the stars, and the winds.*

[1]

Indigenous peoples are resilient peoples with deep traditional knowledge and scientific thought spanning millennia. Indigenous peoples have diverse notions of resilience grounded in culturally distinctive concepts that bridge person, community, and the environment. There is a great importance placed on notions of collective history, the richness of Indigenous languages and traditions, as well as the need for strong individual and collective agency and activism to become a platform for needed change on a global scale [2]. Unfortunately, Indigenous populations have been identified as a highly vulnerable group within global discourse on climate change because of habitation in regions undergoing rapid change, and the disproportionate burden of morbidity and mortality already faced by this population [3]. With Indigenous populations already facing continued and progressive economic and social marginalization, higher prevalence of chronic diseases, and systemic discrimination, the need for Indigenous self-determination and the formal recognition of Indigenous knowledges as a foundation for climate and health solutions specifically are critical [4].

The current and emerging ‘planetary health’ field is most often defined by the ways that humans impact and are impacted by their environment. The notions of air and water quality, food security, and the increasing exposure to natural disasters and infectious diseases are previewed primarily through an anthropocentric lens [5]; however, more recently, a more holistic and inclusive summary of planetary health by Prescott et al. has been put forward in that planetary health, inseparably bonded to human health, is defined as the interdependent vitality of all natural and anthropogenic ecosystems; this vitality includes the biologically defined ecosystems (at micro, meso, and macro scales) that favor biodiversity; it includes the more broadly defined human-constructed social, political, and economic ecosystems that favor health equity and the opportunity to strive for high-level wellness; this definition also includes the business ecosystems that influence sustainable and health-promoting local and global commerce [6].

Regardless, planetary health as a field is primarily a Western construct with Indigenous traditional knowledge (TK) systems having no clear separation between the health of the planet and the health of self or that of the community and ecosystem at large. The long-established interconnected ways of knowing embodied by Indigenous value-systems view social, economic, and ecological aspects as ‘unified systems’ with no divisional aspects apparent [7]. TK itself has been privileged by thousands of years of unified living that is based on a set of Indigenously defined natural laws drawn from the lands, water, and skies that surround us. With this, the path to crisis level current global environmental change (GEC) has been seen from the Indigenous TK perspective to come from the direct appropriation of Indigenous peoples’ ancestral lands. This appropriation subsequently fueled capitalist systems (that work against natural laws) that continue to erode our planet’s capacity as a home for all living things [8]. Land exploitation was and is platformed in most cases with forced societal colonization. This is where Indigenous peoples had Western ways of thinking, believing, acting, trusting, feeling, and living pushed on them, leading only recently to the formal recognition that colonization in and of itself is a determinant of Indigenous health [9]. Colonization continues today through Western modernity with a mismatch occurring between differing environments (i.e., traditional vs modern) through forced relocations, destroyed traditional lands, and loss of access to traditional foods and teachings that take over (i.e., colonize) the molecular processes and structures of Indigenous peoples. These events and processes consequently have detrimental effects on Indigenous health (e.g., higher rates of inflammation [10,11,12], toxic exposures [13,14], and diabetes [15]). 

### Molecular Decolonization: Background and Conceptual Foundations

For Indigenous peoples, the process of ‘decolonization’ (i.e., the countering or outright resistance of colonizing thought, actions, and governance) is based on a framework that centers and privileges Indigenous life, community, and epistemology and is directly rooted in land and country [16]. Indigenous ceremonial practice in particular (which involves an innate connection to land and country) is a deep expression of the decolonization process with physical, mental, emotional, and spiritual connections made from the smallest molecular particle to the largest cosmic galaxy [17]. Understanding that traditional Indigenous knowledges and ceremonial practice span and weave between all scales of existence and the universe underscores the need for a process of decolonization even at the smallest level of being. Colonization (i.e., trauma, genocide, loss of culture and lands, etc.) disrupts Indigenous peoples down to the molecular level with consequent feedforward effects at the planetary level through forced disconnection from our true selves as embodied through nature. In other words, the impacts of colonization alter our behavior, health, relationships, and therefore the health of the planet. The additional continued impacts of macro level consumptive economic systems on all communities, with the consequent entrenchment in poverty, social dysfunction, and reduced quality of life common in much of the world’s population, means we all need to reflectively decolonize from the things that harm us (i.e., monocultured food, sugar, lateral violence, overconsumption, etc.). Molecular decolonization is therefore the process of embracing, restoring, and honoring our original selves at the foundational level of being, in keeping with our original ancestral teachings to inform our future human story (i.e., our original selves, prior to the colonizing mindset, was beset with traditional knowledge and a way of living that positively impacted us and all aspects of the planet down to the molecular level of being which needs to be honored and restored). This molecular decolonization process has the potential to re-balance our biomolecular functioning while restoring the original instructions embedded in our ancestral deoxyribonucleic acid (DNA).

The meaning behind the idea of ‘molecular decolonization’ arose from the Indigenous collective’s engaging in and socializing with the concept through different knowledge traditions and expanding it through formal discourse on a global scale. The term was formally introduced within an Australian Indigenous led cultural public health intervention called the First 1000 Days movement, which was based on the understanding that trauma is carried in our own bodies in addition to that between families generationally [18]. As Indigenous concepts of healing are embodied in our human experience, and because our interconnectivity, ideas, and formulations around molecular decolonization are directly applicable to planetary systems, we are today seeing a form of trauma being embodied in planetary systems that are also in need of healing. With global environmental problems being primarily rooted in injustice and colonization, sustainable long-term solutions will require a paradigm shift away from the reductionist and individualistic approaches common now, towards a paradigm that privileges Indigenous voices and ways of thinking about humanity and our place within the interconnected world [8].

As a group of Indigenous scholars and community members, we emphasize here that ‘molecular decolonization’ is the method back to our original selves, and therefore to our planetary origin story. Molecular decolonization is a strategy that can be used to overcome ‘binarism,’ as it is often expressed in the literature (i.e., ‘Indigenous non-Indigenous,’ ‘Developing-Developed’) [19]. As these binarisms are social and cultural constructions relevant for twentieth-century living, and are not appropriate for the sustainability of our biosphere, strategies that bridge instead of divide are in dire need. We urgently need new civilizational paradigms to rebalance our species’ relationship to all other elements of existence. As a human species we all have the same molecular structure, and therefore, molecular decolonization is a unifying principle to ensure a species response to planetary health and wellbeing.

Through our collective and evolving understanding of decolonization at a molecular level, which many of our Indigenous cultural and healing practices subtly embody, we are better able to translate the intricacies within the current Indigenous scientific worldview through Western forms of discourse. As the health of our molecular being is argued to reflect our planetary being and vice versa (i.e., interconnectedness), the process of molecular decolonization is premised as a strategy for improving environmental health globally. For the most part, we as human beings understand that our eating habits, mental state, and spiritual practices all impact us at a molecular level; however, there is a lack of appreciation in the modern worldview for how those acts and practices impact the planet as an interconnected system. Our health and wellbeing is directly reflected at the planetary scale, and here we provide examples of how the concept of molecular decolonization can be thought about, applied and actioned through a two-eyed seeing lens (i.e., “To see from one eye with the strengths of Indigenous ways of knowing, and to see from the other eye with the strengths of Western ways of knowing, and to use both of these eyes together” [20] in action).

## 2. Going without: Planetary Health and Fasting

The colonization of the hunter-gatherer and traditional diets of Indigenous peoples in North America and elsewhere through the forced adoption of an ad libitum Western diet has been particularly bad for Indigenous peoples and the planet. Before the advent of European agriculture, high-fiber foods such as wild plants, fruits, grasses, seeds, and nuts, along with wild fish and animals were the pillars of the Indigenous diet, undoubtedly producing a superior and diverse gut microbiome [21], along with an excellent omega-three fatty acid profile [22].

Traditional Indigenous horticultural communities produced high quality and nutritionally dense varieties of crops that provided superior health benefits including improved body composition and physical fitness compared with the effects of industrial crops on modern populations [23]. Traditional farming was and is important to planetary health as Indigenous women plant seeds in a reverent, ceremonial manner while treating the plants and earth as possessing sentience (i.e., there is a deep respect for the earth as a living mother). Buffalo Bird Woman, a renowned traditional Hidatsa gardener who was born in 1839, said her people believed that “the corn plants had souls, as children have souls. We cared for our corn in those days, as we would care for a child.” [24]. When spring returned, the women would welcome the corn spirits back from the south with dancing and ceremonies [24]. The traditional relationship with food was therefore complex, deep, and profound. 

Colonization forced Indigenous peoples into a Western lifestyle that resulted in the loss of traditional farming, hunting, fishing, gathering, and cultural practices that many Indigenous communities are fighting to restore and revitalize. This restoration of Indigenous cultural practices is a sign of the continued resilience of Indigenous peoples despite the continued exploitation of Indigenous lands and the large prevalence of adverse social, health, and economic conditions present in communities. Regardless, the Western lifestyle and consequent Western diet has produced “diseases of civilization,’ or what is known in evolutionary biology as “mismatch diseases”. These are diseases that occur because our bodies are poorly or inadequately adapted to the modern environments in which we now live [25]. Cardiovascular disease, obesity, hypertension, type 2 diabetes, cancer, autoimmune disorders, osteoporosis, anxiety and depression, and neurodegenerative conditions such as Alzheimer’s are rare or virtually absent in hunter–gatherers and other non-Westernized populations [23]. Research has suggested that by adopting a diet and lifestyle that mimics the beneficial characteristics of the pre-agricultural environment, the risk of chronic degenerative diseases is then reduced [23], and the stress on the planet is also lessened [26]. Even more recently however, the act of forgoing food itself has been getting more widespread scientific and mass media attention.

Fasting is an overlooked ancient Indigenous practice that has many important implications for human and planetary health. Fasting historically in certain cases was an unplanned event in Indigenous communities during times of food shortage; however, it also played an important role in Indigenous ceremonial life. During unplanned fasting periods, going without food meant not eating or eating sparingly for days or sometimes weeks at a time. To prevent starvation, food was rationed and one ate only when they were truly hungry—demonstrating what Western science now calls “calorie restriction” [27]. It was not uncommon however for Indigenous groups to engage in ceremonial and willful fasting and dancing to gain direction in life, to connect with the natural and spiritual world, to seek permission to utilize the energy from a sacred plant or animal, or even to secure food during a period of starvation in the community. In the winter of 1861, the Mandan and Hidatsa (Indigenous groups from the Great Plains area of the United States) were going through a severe shortage of meat and had very little else to eat. Determined to prevent community starvation, a man named Red Cherry went up on the highest butte outside of the village and fasted for three days to call the buffalo, but he was not successful. The Mandan White Buffalo Cow Society, a women’s organization, stepped in and began dancing day and night and eventually a buffalo appeared [28]. 

Among the Arikara of the Great Plains in the United States, fasting was a requirement for anyone who wished to become a traditional doctor, carry and administer traditional medicines to heal others, and to earn the respect and confidence of the people, ancestors, and the spirits that aided in the doctoring (i.e., healing) process. Ella P. Waters (Yellow Bird Woman), who was born in 1889 on the Fort Berthold Reservation in North Dakota, was a highly respected Arikara woman and the last traditional doctor in her community. As a young woman on her way to becoming a traditional doctor, she looked after the elderly traditional doctors in the sacred Medicine Lodge, taking them food, attending to their needs, and manifesting what she described as “holy feelings” [29]. At age eighteen, “she began going to the Medicine Lodge to fast, make sacrifices of food and goods, and ask the doctors’ societies for blessings” [29]. Ella passed away in 1984 at age ninety-five.

Unfortunately, there are fewer and fewer Indigenous groups today regularly engaging in traditional farming or ritual fasting. Recently however, there has been great effort to change this, with a greater push from traditional Indigenous leaders and traditional medicine people to support a reconnection with Indigenous culture and ceremony. The revitalization of fasting has gained more widespread acknowledgement in the Western scientific community with new data pointing towards favorable molecular benefits from engaging in this practice. Research has evaluated calorie restriction [30], alternate day eating [31], and periodic fasting to reverse various adverse health effects [32] and improve metabolic markers [33,34]. Of the fasting approaches studied, the twelve- to twenty-one-hour intermittent fast (IF) has had the highest compliance by individuals habituated to the Western lifestyle [35]. Studies on IF in animals as well as human clinical trials have demonstrated the lowering of biomarkers associated with chronic illness, including LDL cholesterol and triglycerides [33,34], fasting blood glucose and insulin levels [33,34], and C-reactive protein (CRP) and Tumor necrosis factor alpha (TNF-𝞪) [33,34].

At the molecular level, caloric restriction induces oxidative stress on the mitochondria, triggering autophagy, the cellular mechanism for organelle (specialized structures within cells) quality control [36]. Lysosomes (a type of organelle) then devour these damaged cellular structures and therefore mediate apoptosis (i.e., programmed cell death) [37]. The metabolic stress noted and induced by the intermittent fasting state helps to maintain autophagic flux, which is the cellular process of degrading and recycling excess or damaged cell structures [36,38]. Dysregulated autophagy is a feature of mismatch diseases (i.e., diseases that occur because our bodies are poorly or inadequately adapted to environments in which we now live), whereas healthy cell metabolism is a continuous balance of anabolism and catabolism, build-up and breakdown that helps to maintain health [32]. Therefore, at the cellular level, growth and replication is tempered by autophagy and apoptosis, mirroring cycles of life and death on our planet. A planetary microcosm, so to speak.

We human beings require tempered patterns of consumption in reciprocity with the land to maintain balance (like healthy autophagic flux). Colonialism and the Western lifestyle is described by the Indigenous Ojibwe word ‘windigo’, highlighting overconsuming gluttony and greed [39]. With this, the phrase ‘evolutionary mismatch’ could be used to describe not only the human health effects of the Western lifestyle, but also its severed, extractive relationship to the earth. A lifestyle of dietary overconsumption wreaks havoc on the land and on our more-than-human relatives from the loss of ecosystem diversity (e.g., clearcutting for cattle ranching, loss of soil nutrients through monocrop agriculture, and the loss of beneficial microbes in the soil from the use of pesticides). The health of the land is mirrored by the current state of our inner terrain (i.e., the intestinal microbiome), which has been compromised from eating processed, refined foods leaving a trail of destruction. Fasting on the other hand has been found to increase the diversity of intestinal flora compared to the current on-demand feeding schedule that is common in our modern society, which consequently reduces its and our complexity in the process [40]. As with most ecosystems, richer diversity is a reflection of more robust health. A diverse microbiome shapes the innate immune response, supporting the function of T regulatory immune cells and conferring immunoprotection [38], just as ecological diversity is so important for our planet’s wellbeing and protection. 

So, by reducing individual food consumption in a healthy way, divesting from industrialized food systems, and fasting, we can have a decolonial impact on the planet by reducing our carbon footprint and helping the land and humans to heal right down to the molecular level of being. This reclamation of ancestral foodways, teachings, and ceremonial practice helps us to remember our rooted relationship and responsibility to Mother Earth and the next seven generations that come after us (i.e., the ‘seven generation principle’ [41]). Restoring Indigenous sovereignty and practice around food is a crucial component to decolonization, which “brings about repatriation of Indigenous land and life” [42].

## 3. A Micro- to Meta-Narrative: Utilizing Molecular Decolonization Concepts in Time-Sensitive Movements

Planetary health movements recognize the world has a unique but fast-closing window of opportunity to safeguard our future [43]. Like planetary health advocates, implementers of the international First 1000 Days movement have focused on time-sensitive interventions to realize lifelong health and wellbeing [44]. The First 1000 Days movement uses the time period from conception until a child’s second birthday to build healthier and more prosperous futures [45]. Both planetary health and the First 1000 Days movements are founded in sciences demonstrating how internal and external environments—our in utero experiences, the food we eat, the air we breathe, our access to fresh water, and the energy we use—shape our future health in significant ways.

Although there is envisaged symmetry between the conceptualizations needed to unify our human community around a set of specific calls to action to improve health outcomes from birth onwards for all, there is little evidence to illustrate how our common microbial and molecular communities are being used to inform narratives that unite all life on Earth. The foundation of these developing microbial-based decolonizing narratives are premised on the need for rebalancing our biomolecular functioning within and across species while dampening the supercilious viewpoint that human health should be put ahead of all others on Earth. 

To decolonize this human-centric worldview and create renewed Earth-centered communities spanning generations (‘all my Ancestors’), and places (migration and seasonal life and living), an Indigenous strategy to refocus global narratives by enhancing the vitality of our microbial communities is warranted. The microbial microcosm is a compelling narrative that situates our human biome in the biome of the planet, and in doing so, provides a common language to bridge efforts across and between movements, humans, and our natural environments. Current Indigenous-led initiatives recognize microbial communities as having sentient purpose with diverse habitats represented from the soil to the human body. These complex ecological interactions and relationships with each other, the environment, and across generations, is a solid foundation upon which to create a unifying meta-narrative for our time [46].

Microbiome science itself is increasingly recognized within the planetary health and the First 1000 Days movements [47]. Through a planetary health lens, microbiome science underscores the importance of accumulated experiences within the total lived environment linking biological, psychological, and social equations critical to personal, public, and planetary scales [48]. Relevant to the First 1000 Days movement is the understanding and appreciation for the intergenerational impact of molecular interactions in the human genome and microbiome. For example, epigenetics research demonstrates that exposures to adverse experiences in utero (including inadequate or altered nutrition, environmental toxins, abusive behaviors, and social stresses) produce genomic changes that can be transmitted across generations and contribute to the non-genomic transmission of disease [49] (i.e., induce a state of imbalance). On the other hand, encouraging healthy microbial communities has been recognized to provide intergenerational resilience [50] (i.e., there is balance and harmony with nature). 

Resilience in human, microbial, and greater ecological communities is, quite simply, the amount of stress that can be tolerated before a living system’s trajectory changes toward a different equilibrium state [51,52]. At the macrosystem level, studies illustrate how human-driven pollution, land usage, and resource exploitation erode ecological systems into less productive states, making them less resilient overall. Similarly, studies of global increases in temperature and climatic instability demonstrate how stress impacts the micro-level molecular mechanisms regulating plant, animal, and human growth and development [53], therefore also decreasing their overall resiliency. Current Indigenous knowledge systems have used micro-level communities (now modernly termed ‘microbial communities’) to characterize acts and practices that promote resilience and connect humans to macro-planetary systems (such as the laying down of a newborn baby on the Land in microbial-rich soil after birth to establish connection). So, although modern microbiome science is an emerging field in some knowledge traditions, Indigenous peoples’ science systems demonstrate a propensity for understanding molecular communities, which is evidenced by the purposeful genetic manipulation through cropping, agricultural practices, and ecosystem maintenance [54], in addition to certain medicinal and ceremonial practices. This laying down of a baby on land and country in ceremony upon birth demonstrates a powerful reverence and awareness for microbial communities that ensures a direct facilitation of microbial exchanges between mother, child, and Mother Earth. In a story collected from central Australia over two decades ago, these microbial exchanges were a feature of a child’s birth and connected them to the ecosystems in which they were born:

In the old days, tribal way, we had no medicine. We had no woman trouble, we used to put hot ashes on our tummies and back when we born those babies. They were not born with blanket; they were born straight into the ground. When that baby was born, we would dig a hole in the ground and put green leaves and branches in the hole. We would smoke those babies and ourselves to make us strong. We would put black ochre across our baby boy’s forehead, and on our daughters, we would put black ochre on their stomachs. We would put hair string around their necks and stop them from crying too much.

[55]

In an Australian context and in contemporary times, this connection to country is provided through an ‘Acknowledgement of Country’ in which respect is paid to the traditional owners of the land. Elders acknowledge visitors to their lands as a way of offering safe passage and protection of the visitor’s spiritual wellbeing during their birth journey. Reintroduced decolonized cultural practices, such as the ‘Welcome Baby to Country’ in the First 1000 Days Australia movement, is gaining popularity once again. These ceremonies include babies being smoked and given ancestral names by the community, while directly involving parents in practices that connect people to country—through carving wood baby carriers, taking children to be cleansed in rivers, and being ‘ochered’ by Elders. 

As humans, our first ecologic experience is that of our mother’s womb. Our long-term health and wellbeing are dependent on the qualities of the exchange of essential nutrients and bacteria from our mothers during pregnancy [56]. Birthing is a process that then brings us into our second ecologic experience. This early transition to our second ecological lived experience is defined by the qualities and characteristics of the social, cultural, structural, and other supports available to the mother during her child’s early life. These shared, early-life microbial and ecologically rooted experiences between mother and baby are defined by our humanity and shared with other organisms through our interconnected existence. Microbial communities are therefore germane to our planet’s communities of life and are a part of our origin story.

Decolonized narratives that are centered on the molecular structuring of the world are relevant to Indigenous peoples and advocated for by many others who draw inspiration from Earth-centered cultural traditions and governance systems. As the genesis of ‘molecular decolonization’ is derived from within Indigenous knowledge systems, the use of this term not only recognizes the unique sensitivities of Indigenous peoples to climate emergencies, but also repositions the contribution of Indigenous peoples’ knowledge as central to twenty-first-century living [57]. Indigenous knowledge is essential to the crafting of alternative narratives for life on Mother Earth. Movements such as planetary health and the First 1000 Days promote scalable mitigation models that benefit human health by connecting health and wellbeing to that of ecosystem health [58] (micro to macro). In these movements, ‘molecular decolonization’ as a concept can be embedded in a meta-narrative of ‘unified microbial communities’ (i.e., the interconnectedness between all things) that have the metaphoric potential to relinquish twentieth-century reliance on fossil fuels and encourage the embrace of Earth-centered jurisprudence as necessary for future life to occur on Earth. Molecular decolonization is part of a meta-narrative that uses the smallest scale of life on Earth to connect to the macro while disrupting the power relations that see Indigenous knowledge systems outside of, less than, or trivial by those humans and systems heavily invested in Western knowledge constructs. 

Earth-centered jurisprudence systems govern both microbial and global systems (i.e., there is a unity of natural laws). Earth-centered jurisprudence systems can also be utilized to reinforce harmonization of macro-financial and fiscal policies (while protecting against the fragmentation of initiatives), which consequently have the potential to lead to a reduction in carbon emissions, an increase in bio-diversity, and an ability to support nations in preparing for and adapting to the amplification of climate emergencies that cause widespread harm and damage to livelihoods [59]. In the vision for a healthy future, it is important to create the conditions that enable the overcoming of the dissonance between ‘being in nature’ (i.e., nature that surrounds us) and ‘being of nature’ (i.e., nature that embodies us). Words as concepts and experiences matter as they shape our thinking about the world and, in turn, the actions we take [60].

## 4. Decolonizing Stories in Planetary Health: From Micro to Macro

The cultural practices and stories that Indigenous peoples have retained and continue to perpetuate have direct impacts on our overall health and wellbeing [61]. By engaging and embodying our ancestral teachings and practices told through our stories, we consequently reduce the stress on our molecular and planetary environment, as the teachings are premised on a deep respect for land and country (i.e., Mother Earth connectivity) through decolonizing narratives. The impact of storytelling as a formal teaching methodology for planetary and public health is an underestimated and underutilized science in the quest to regain our planetary balance [62]. So, in keeping with an Indigenous research methodology [63] and style of scholarship that has spanned countless generations, we here share space for intricate story by Tyson Yunkaporta, a member of the Apalech Clan in the far north of Queensland, Australia:

In saltwater country our old people pass on knowledge of all things biotic, things of spirit and flesh, micro and macro, but not divided into those alien categories. The knowledge is in-between, like the vast spaces between atoms or stars. The aerial roots of mangroves that draw gases into those plants have the same name in language as the word for lungs, the word and the phenomenon it names both grounded in ancient Lore that indicates an unseen world of minute relations beyond the reach of the eye.

In saltwater it is known that there are tiny entities that are friendly and may enter through the skin and become part of us, invigorating our bodies and making us part of our coastal bioregion, down deep in our bones, patterned and sung. It is the same transformative action that enables eels to change from freshwater to saltwater species in cycles of migration throughout their lives.

A scientist looking at these aquatic viruses will tell us they are plant beings, but when they say phyto we might think “Fight-o!” until we read the research paper and recognize them as the little helpers we know so intimately, symbiotic green things that form basins of attraction in the co-evolutionary fitness surfaces of our complex bodily systems. There are both plant and animal viruses, and they work with us in healthy systems right up to the point at which our systems are knocked out of balance when they then work against us. This occurs at both micro and macro levels.

When ‘country’ is sick, there are co-morbidities that arise as plants and animals adapt in crazy ways, responding to insane new patterns of entropy that are eating the land alive. There is die-back in the mangroves in “untouched wilderness” areas and nobody knows why they are choking to death in the dry season, or why our old people die in their sleep during flu season at the same time. Pathogens are thrown up by animal refugees hopping and flapping and swimming away from the environmental carnage, seeking safer destinations in vain. In veins, smaller things repeat that pattern, replicating wildly and searching for new habitats across species, including humans.

I am thinking of this as I watch my babies play with a spiky rubber ball in our backyard. Their hands are sticky with honey from their breakfast toast, making it hard to throw that ball. I think of that animal virus and its spiky skin that is attracted to proteins on the surface of our cells. I think of the protein and cellulose taxonomy of our Indigenous universe, reflected in our language and our dietary habits. We put the protein suffix in front of the names for beings with blood in their veins, and the cellulose suffix in front of the names for beings with sap in their veins. We balance these two food groups in our diet, as the protein-based foods have bio-available nutrients for our bodies, while the cellulose-based foods have bio-available nutrients for the ecosystem of tiny creatures that live within our gut.

That gut is our center of power, a separate neural system that operates independently of our brains. The tiny creatures in there act like neurons, and the gut, as the seat of our big spirit, steers the course of our lives instinctively and ancestrally. We inherit that gut flora from our mother in the womb and if we have access to undomesticated foods in that dynamic balance of protein and cellulose from time to time, and we do not ingest too much poison, we live free of the auto-immune diseases, autism, and cancers that have become plagues in the last few decades. We have been free of these things for millennia, living also in harmony with the helpful viruses that move through land and body systems existing in a state of balance and complex relations.

So, my babies laugh and play with that spiky ball that keeps sticking to their snot-and-honey-slicked hands, and I think of the sugarbag, the native honey that we are missing as we camp here in this cold southern city. Those little black bees that make the honey exist in a sub-equatorial belt around the globe, in South America and South Africa as well as here. They do not like crossing saltwater—maybe that big manta ray will swim up and leap out of the waves to drown them. Their hives are not the regimented yellow grids of domesticated European and Asian bees—the wax is black and formed in a sacred energetic spiral. 

There is both pollen and honey in there, and in language the pollen takes the protein suffix and the honey takes the cellulose suffix. The honey regulates the activity of bacteria and the pollen regulates the activity of viruses. A drink made of either can heal the diseases that form when systems are knocked out of balance and the tiny entities inside our bodies fall out of symbiotic relation (or, more often, when we fall out with them). 

There is nothing pathological about these tiny relatives of ours that have recently been colonized and misnamed as pathogens, framed as the invisible enemy of a dying empire currently occupying our lands and waging its endless wars. That is the macro conflict. When we are ill, we go through micro conflicts, internal struggles, but our tiny relations inside are not the enemy—they are helping us to learn and grow, to transform—even if that lesson means it is time to move between worlds or into new epochs. 

Time is an important variable here in the patterns that make changes between micro and macro. That sugarbag is only medicinal in the right season when you see the yellow flowers blooming. If your lungs are still out of balance after that (maybe a bit too much pollen about) then you might eat a nice fat bat in that season to fix your breathing. Still, you will only breathe as well as the mangroves are breathing on your country, because you are an organism in dynamic, co-evolutionary relation with its habitat. The spirit of mangrove, flower, honeybee, and bat is moving through you and you are moving through it, whether your markets are wet or dry, booming or busting.

Whether spirit exists in the Western sense of the word or not, it is the heuristic we use as Indigenous people in a complex system of knowledge of macro and micro worlds, patterned on millennia of relational existence within a dynamic landscape that holds all of that rich knowledge. Whether your lens is situated in a totemic system or a microscope, this lore is vital to understanding the microbial universe, from the quantum level to the galactic. Decolonizing the microbiome must therefore involve the incorporation of appropriate lore in the ongoing process of inquiry that our survival demands in periods of imbalance and transition.

## 5. Conclusions

The planet’s health is a reflection of our own health and wellbeing right down to the molecular level. When Mother Earth is sick and unbalanced, we are also sick and unbalanced. Due to the interconnected web of our existence, there is great need to reconsider our daily practices and thought processes through the lens of reciprocity, responsibility, and relationality to our Mother Earth. Through the process of reconnecting to our original selves (our true origin), we go through a process of decolonization that reforms us at the molecular level (i.e., molecular decolonization). Our ancestral bodies are directly rooted in the land and country where we come from (i.e., our foundational level of being), and are capable of remembering our connection to all things when we give it the space to do so. Our planet’s health and very existence depends on our remembering where we came from.

## References

[1] Redvers N. (2018). The Value of Global Indigenous Knowledge in Planetary Health. Challenges.

[2] Kirmayer L.J., Dandeneau S., Marshall E., Phillips M.K., Williamson K.J. (2011). Rethinking Resilience from Indigenous Perspectives. Can. J. Psychiatry.

[3] Ford J.D. (2012). Indigenous Health and Climate Change. Am. J. Public Health.

[4] Jones R. (2019). Climate change and Indigenous Health Promotion. Glob. Health Promot..

[5] Myers S.S. (2017). Planetary health: Protecting human health on a rapidly changing planet. Lancet.

[6] Prescott S., Logan A., Albrecht G., Campbell D., Crane J., Cunsolo A., Holloway J., Kozyrskyj A., Lowry C., Penders J. (2018). The Canmore Declaration: Statement of Principles for Planetary Health. Challenges.

[7] Sangha K.K., Le Brocque A., Costanza R., Cadet-James Y. (2015). Ecosystems and indigenous well-being: An integrated framework. Glob. Ecol. Conserv..

[8] Ratima M., Martin D., Castleden H., Delormier T. (2019). Indigenous voices and knowledge systems—Promoting planetary health, health equity, and sustainable development now and for future generations. Glob. Health Promot..

[9] Jones R., Crowshoe L., Reid P., Calam B., Curtis E., Green M., Huria T., Jacklin K., Kamaka M., Lacey C. (2019). Educating for Indigenous Health Equity. Acad. Med..

[10] Kheir J.M., Guthridge C.J., Johnston J.R., Adams L.J., Rasmussen A., Gross T.F., Munroe M.E., Bourn R.L., Sivils K.L., Guthridge J.M. (2018). Unique clinical characteristics, autoantibodies and medication use in Native American patients with systemic lupus erythematosus. Lupus Sci. Med..

[11] Hodge A.M., Maple-Brown L., Cunningham J., Boyle J., Dunbar T., Weeramanthri T., Shaw J., O’Dea K. (2010). Abdominal obesity and other risk factors largely explain the high CRP in Indigenous Australians relative to the general population, but not gender differences: A cross-sectional study. BMC Public Health.

[12] Scofield R.H., Sharma R., Pezant N., Kelly J.A., Radfar L., Lewis D.M., Kaufman C.E., Cioli S., Harris J., Grundahl K. (2019). American Indians Have A Higher Risk Of Sjögren’s Syndrome And More Disease Activity Than Caucasians And African-Americans. Arthritis Care & Research.

[13] Dashner-Titus E.J., Hoover J., Li L., Lee J.H., Du R., Liu K.J., Traber M.G., Ho E., Lewis J., Hudson L.G. (2018). Metal exposure and oxidative stress markers in pregnant Navajo Birth Cohort Study participants. Free Radic. Biol. Med..

[14] Lewis J., Hoover J., MacKenzie D. (2017). Mining and Environmental Health Disparities in Native American Communities. Curr Environ. Health Rep..

[15] Cho P.G.L., Burrows N.R., Roberts D.L., Bullock A.K., Toedt M.E. (2014). Diabetes-related mortality among American Indians and Alaska Natives, 1990-2009. Am. J. Public Health.

[16] Sium A., Desai C., Ritskes E. (2012). Towards the ‘tangible unknown’: Decolonization and the Indigenous future. Decolonization Indig. Educ. Soc..

[17] Redvers N. (2019). The Science of the Sacred: Bridging Global Indigenous Medicine Systems and Modern Scientific Principles.

[18] Arabena K. Molecular Decolonization Lecture. Proceedings of the First 1000 Days Australia Summit.

[19] Germond-Duret C. (2016). Tradition and modernity: An obsolete dichotomy? Binary thinking, indigenous peoples and normalisation. Third World Q..

[20] Bartlett C., Marshall M., Marshall A. (2012). Two-Eyed Seeing and other lessons learned within a co-learning journey of bringing together indigenous and mainstream knowledges and ways of knowing. J. Environ. Stud. Sci..

[21] Clemente J.C., Pehrsson E.C., Blaser M.J., Sandhu K., Gao Z., Wang B., Magris M., Hidalgo G., Contreras M., Noya-Alarcón Ó. (2015). The microbiome of uncontacted Amerindians. Sci. Adv..

[22] Stark K.D., Van Elswyk M.E., Higgins M.R., Weatherford C.A., Salem N. (2016). Global survey of the omega-3 fatty acids, docosahexaenoic acid and eicosapentaenoic acid in the blood stream of healthy adults. Prog. Lipid Res..

[23] Carrera-Bastos P., Fontes-Villalba M., O’Keefe J., Lindeberg S., Cordain L. (2011). The western diet and lifestyle and diseases of civilization. Res. Rep. Clin. Cardiol..

[24] Gilman C., Schneider M.J. (1987). The Way to Independence: Memories of a Hidatsa Family, 1840–1920.

[25] Daniel L. (2013). The Story of the Human Body: Evolution, Health, and Disease.

[26] Willett W., Rockström J., Loken B., Springmann M., Lang T., Vermeulen S., Garnett T., Tilman D., Declerck F., Wood A. (2019). Food in the Anthropocene: The EAT–Lancet Commission on healthy diets from sustainable food systems. Lancet.

[27] Lee C., Longo V. (2016). Dietary restriction with and without caloric restriction for healthy aging. F1000Research.

[28] Fenn E.A. (2015). Encounters at the Heart of the World: A History of the Mandan People.

[29] Parks D.R. (1991). Traditional Narratives of the Arikara Indians-Stories of Alfred Morsette: English Translations.

[30] Wei M., Brandhorst S., Shelehchi M., Mirzaei H., Cheng C.W., Budniak J., Groshen S., Mack W.J., Guen E., Di Biase S. (2017). Fasting-mimicking diet and markers/risk factors for aging, diabetes, cancer, and cardiovascular disease. Sci. Transl. Med..

[31] Trepanowski J.F., Kroeger C.M., Barnosky A., Klempel M.C., Bhutani S., Hoddy K.K., Gabel K., Freels S., Rigdon J., Rood J. (2017). Effect of Alternate-Day Fasting on Weight Loss, Weight Maintenance, and Cardioprotection Among Metabolically Healthy Obese Adults. JAMA Intern. Med..

[32] Mattson M.P., Longo V.D., Harvie M. (2017). Impact of intermittent fasting on health and disease processes. Ageing Res. Rev..

[33] Patterson R.E., Sears D.D. (2017). Metabolic Effects of Intermittent Fasting. Annu. Rev. Nutr..

[34] Antoni R., Johnston K.L., Collins A.L., Robertson M.D. (2017). Effects of intermittent fasting on glucose and lipid metabolism. Proc. Nutr. Soc..

[35] Wegman M.P., Guo M.H., Bennion D.M., Shankar M.N., Chrzanowski S.M., Goldberg L.A., Xu J., Williams T.A., Lu X., Hsu S.I. (2015). Practicality of Intermittent Fasting in Humans and its Effect on Oxidative Stress and Genes Related to Aging and Metabolism. Rejuvenation Res..

[36] Liu H., Javaheri A., Godar R.J., Murphy J., Ma X., Rohatgi N., Mahadevan J., Hyrc K., Saftig P., Marshall C. (2017). Intermittent fasting preserves beta-cell mass in obesity-induced diabetes via the autophagy-lysosome pathway. Autophagy.

[37] Antunes F.E.A., Costa A.J., Nascimento A.C., Bincoletto C., Ureshino R.P., Pereira G.J.S., Smaili S.S. (2018). Autophagy and intermittent fasting: The connection for cancer therapy?. Clinics (Sao Paulo).

[38] Traba J., Kwarteng-Siaw M., Okoli T.C., Li J., Huffstutler R.D., Bray A., Waclawiw M.A., Han K., Pelletier M., Sauve A.A. (2015). Fasting and refeeding differentially regulate NLRP3 inflammasome activation in human subjects. J. Clin. Investig..

[39] Forbes J.D. (2008). Columbus and Other Cannibals: The Wetiko Disease of Exploitation, Imperialism and Terrorism.

[40] Cignarella F., Cantoni C., Ghezzi L., Salter A., Dorsett Y., Chen L., Phillips D., Weinstock G.M., Fontana L., Cross A.H. (2018). Intermittent Fasting Confers Protection in CNS Autoimmunity by Altering the Gut Microbiota. Cell Metab..

[41] 7th Generation Principle. http://7genfoundation.org/7th-generation/.

[42] Tuck E., Yang K.W. (2012). Decolonization is not a metaphor. Decolonization Indig. Educ. Soc..

[43] Whitmee S., Haines A., Beyrer C., Boltz F., Capon A.G., De Souza Dias B.F., Ezeh A., Frumkin H., Gong P., Head P. (2015). Safeguarding human health in the Anthropocene epoch: Report of The Rockefeller Foundation–Lancet Commission on planetary health. Lancet.

[44] Why 1000 Days. https://thousanddays.org/why-1000-days/.

[45] Building Health. https://thousanddays.org/why-1000-days/building-health/.

[46] Konopka A. (2009). What is microbial community ecology?. ISME J..

[47] Armenteras D., Lavorel S., Lengyel S., Scholes B., Santos-Martin F., Biggs R., Brink B., Koleff P., Henle K., Cramer W. (2016). Chapter 2: IPBES Assessments Across Scales. Guide on the Production and Integration of Assessments from and Across All Scales (deliverable 2 (a)). IPBES/4/INF/9.

[48] Prescott S.L., Wegienka G., Logan A.C., Katz D.L. (2018). Dysbiotic drift and biopsychosocial medicine: How the microbiome links personal, public and planetary health. BioPsychoSoc. Med..

[49] Yehuda R., Lehrner A. (2018). Intergenerational transmission of trauma effects: Putative role of epigenetic mechanisms. World Psychiatry.

[50] Ling Y., Watanabe Y., Okuda S. (2019). The Human Gut Microbiome is Structured to Optimize Molecular Interaction Networks. Comput. Struct. Biotechnol. J..

[51] Lozupone C.A., Stombaugh J.I., Gordon J.I., Jansson J.K., Knight R. (2012). Diversity, stability and resilience of the human gut microbiota. Nature.

[52] Folke C., Carpenter S., Walker B., Scheffer M., Elmqvist T., Gunderson L., Holling C.S. (2004). Regime Shifts, Resilience, and Biodiversity in Ecosystem Management. Annu. Rev. Ecol. Evol. Syst..

[53] Ding Y., Shi Y., Yang S. (2020). Molecular Regulation of Plant Responses to Environmental Temperatures. Mol. Plant..

[54] Pascoe B. (2014). Dark Emu Black Seeds: Agriculture or Accident?.

[55] Arabena K. (1996). ‘Risky Business Workshop Facilitator’s Manual’ Tristate Project.

[56] Stinson L.F., Boyce M.C., Payne M.S., Keelan J.A. (2019). The Not-so-Sterile Womb: Evidence That the Human Fetus Is Exposed to Bacteria Prior to Birth. Front. Microbiol..

[57] Arabena K. (2006). The Universal Citizen: An Indigenous Citizenship Framework for the Twenty-first Century.

[58] Reyes-García V., Fernández-Llamazares Á., McElwee P., Molnár Z., Öllerer K., Wilson S.J., Brondizio E.S. (2019). The contributions of Indigenous Peoples and local communities to ecological restoration. Restor. Ecol..

[59] Arabena K., Armstrong F., Berry H., Brooks P., Capon T., Crabb B., Demaio A., Doherty P., Lewin S., Lo S. (2018). Australian health professionals’ statement on climate change and health. Lancet.

[60] Blythe J., Daigle C., Baird J. The Meaning of Environmental Words Matter in the Age of ‘Fake News’. https://theconversation.com/the-meaning-of-environmental-words-matters-in-the-age-of-fake-news-106050.

[61] Kingsley J., Munro-Harrison E., Jenkins A., Thorpe A. (2018). “Here we are part of a living culture”: Understanding the cultural determinants of health in Aboriginal gathering places in Victoria, Australia. Health Place.

[62] Datta R. (2018). Traditional storytelling: An effective Indigenous research methodology and its implications for environmental research. AlterNativ. Int. J. Indig. Peoples.

[63] Wilson S. (2001). What Is an Indigenous Research Methodology?. Can. J. Nativ. Educ..

